# Aptamers provide superior stainings of cellular receptors studied under super-resolution microscopy

**DOI:** 10.1371/journal.pone.0173050

**Published:** 2017-02-24

**Authors:** Maria Angela Gomes de Castro, Claudia Höbartner, Felipe Opazo

**Affiliations:** 1 Institute of Neuro- and Sensory Physiology, University Medical Center Göttingen, Göttingen, Germany; 2 Institute for Organic and Biomolecular Chemistry, Georg-August-University, Göttingen, Germany; 3 Center for Biostructural Imaging of Neurodegeneration (BIN), University of Göttingen Medical Center, Göttingen, Germany; Consiglio Nazionale delle Ricerche, ITALY

## Abstract

Continuous improvements in imaging techniques are challenging biologists to search for more accurate methods to label cellular elements. This is particularly relevant for diffraction-unlimited fluorescence imaging, where the perceived resolution is affected by the size of the affinity probes. This is evident when antibodies, which are 10–15 nm in size, are used. Previously it has been suggested that RNA aptamers (~3 nm) can be used to detect cellular proteins under super-resolution imaging. However, a direct comparison between several aptamers and antibodies is needed, to clearly show the advantages and/or disadvantages of the different probes. Here we have conducted such a comparative study, by testing several aptamers and antibodies using stimulated emission depletion microscopy (STED). We have targeted three membrane receptors, EGFR, ErbB2 and Epha2, which are relevant to human health, and recycle between plasma membrane and intracellular organelles. Our results suggest that the aptamers can reveal more epitopes than most antibodies, thus providing a denser labeling of the stained structures. Moreover, this improves the overall quality of the information that can be extracted from the images. We conclude that aptamers could become useful fluorescent labeling tools for light microscopy and super-resolution imaging, and that their development for novel targets is imperative.

## Introduction

Conventional light microscopy has a resolution limit imposed by the diffraction of light. In practical terms, small elements that are closer than ~200 nm from each other cannot be detected as separate features. Currently, several methodologies are able to surpass the limit imposed by the diffraction of light [1,2]. Diffraction unlimited microscopes are improving very quickly, and to date excellent resolutions can be attained (<10 nm) [3]. However, the improvement of sample preparation and staining methodologies is lagging behind. For instance traditional immunostaining techniques rely on affinity tools that are sometimes larger than the protein of interest, and the full potential of modern imaging techniques cannot be exploited. In fact, the primary–secondary antibody complex of traditional immunostaining techniques can be up to 25 nm long, which is not only larger than some resolution limits of today’s instruments but also results in low density of labeling due to steric hindrance [1,4]. Therefore, it is expected that small probes might help to improve the staining precision on biological samples. Recently, it has been suggested that small single domain antibodies (sdAb or nanobodies) are able to position the fluorescent molecules closer to intended target, resulting in improved localization accuracies when compared to conventional antibody stainings in super-resolution microscopy [5,6]. Similarly, aptamers have been also proposed as an alternative small probe with comparable advantages in the field of super-resolution microscopy [7].

Aptamers are single-stranded DNA or RNA oligonucleotides with lengths ranging from 15 to 100 nucleotides [8]. The aptamers’ nucleotide sequence determines their three-dimensional structure that provides the specific binding to the target molecules [8]. Aptamers are typically generated *in vitro* by a process called systematic evolution of ligands by exponential amplification or SELEX [9,10].

Aptamers have been selected against a large variety of targets, including ions [11,12], small organic molecules [8,13,14], whole cells [15,16] and viruses [17–19]. However, their use in imaging and super-resolution microscopy has not been sufficiently characterized and exploited. Therefore, a comparative study with conventional staining methods to test multiple aspects of the binding and imaging abilities of aptamers are of great importance for the future development and application of aptamers as imaging tools. In this study, we have used stimulated emission depletion (STED) microscopy to systematically compare the staining characteristics of three commercially available aptamers against various antibodies. All aptamers used in this study were synthesized with the chemical modification 5-(*N*-benzylcarboxyamide)-2′-deoxyuridine (called 5-BzdU) replacing the standard thymidine nucleoside. Previous studies suggested that the presence of hydrophobic groups in aptamers like benzyl, pentynyl, napthyl or indolyl result in aptamers with stronger binding affinities (or smaller *k*_off_) to their targets [20–22]. The selected target proteins for the study were the epidermal growth factor receptor (EGFR), the human epidermal growth factor receptor 2 (ErbB2; also known as HER2) and the ephrin type-A receptor 2 (Epha2). We observed that aptamers tend to find more epitopes and provide therefore higher labeling densities than antibodies, which resulted in better definition of the imaged subcellular structures. The increasing amounts of commercially available aptamers will make easier for scientist to choose the right tagging system for their protein of interest, especially if using super-resolution microscopy techniques.

## Results

### The EGFR, ErbB2 and Epha2 aptamers provide highly specific stainings

**Fig 1:**
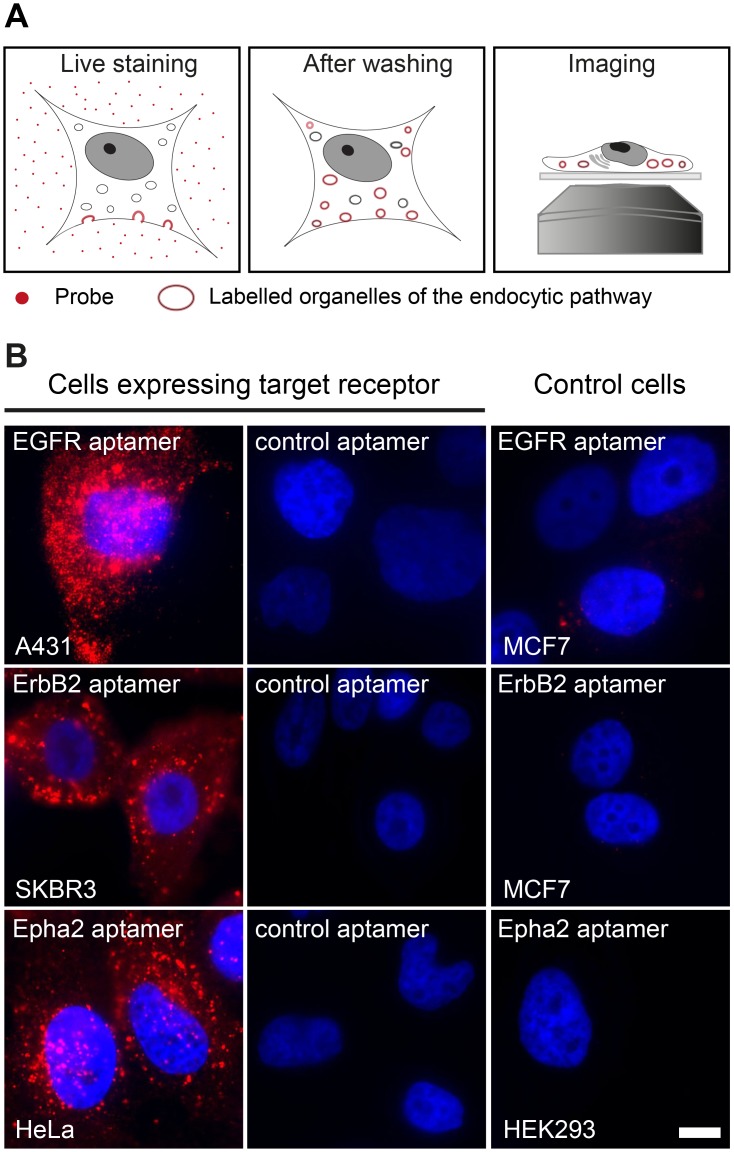
Live staining and aptamer specificity. (**A**) Simple scheme describing the procedure to stain living cells using aptamers or antibodies (probes). Cells are incubated with aptamer or antibodies allowing the internalization of the probes by the endocytosis of the receptor. (**B**) Aptamers against EGFR, ErbB2 and Epha2 were used to live-stain human cell lines expressing these receptors (A431, SKB3 and HeLa cells respectively). A control aptamer with randomized sequence of equivalent length and coupled to the same fluorophore was used to stain these cell lines. Additionally, cell lines that do not express such receptors were used as negative control cells (MCF7 cells are negative for EGFR and ErbB2; HEK293 cells are negative for Epha2). Both controls, the cell lines not expressing the receptor and the randomized aptamer show virtually no aptamer signal (nuclei were DAPI stained, displayed in blue). All images were acquired using an epifluorescence microscope with the same settings and they are equally scaled to allow a direct comparison. Scale bar represents 5 μm.

Since all epitopes in this study are on the extracellular portion of the receptors, aptamer and antibody stainings were performed on living cells. To avoid exogenous overexpression of the receptor of interest (i.e. via plasmids or viruses) we used different human cell lines that were characterized to have detectable endogenous expression of the receptors of interest. We used A431 cells for EGFR [23], SKBR3 for ErbB2 [24] and HeLa for Epha2 [25]. We then exposed living cells to fluorescent aptamers (an later also to antibodies) for a relatively prolonged amount of time (60 min) to maximize the labeling of the endocytic and trafficking pathway for each specific receptor (e.g. early & late endosomes, lysosomes, etc, scheme in Fig 1A) [7,26,27]. This labeling pattern was confirmed by the appearance of fluorescently labeled organelles or endosome-like structures observed along the cytoplasm after staining with saturating concentrations of all tested aptamers (Fig 1B). Importantly, the staining of the same cells expressing the target receptor but using a randomized aptamer sequence (control aptamer) resulted in almost undetectable signal. Similarly, the staining of cells not expressing the particular receptor (control cells) using the specific aptamers at the same concentrations displayed no detectable signal (Fig 1B). These results strongly suggest that the commercially available aptamers used in this study are indeed specific for their intended targets and that coupling them to a fluorescent molecule does not impair their binding ability or their specificity.

### The aptamers label endosome-like organelles

**Fig 2:**
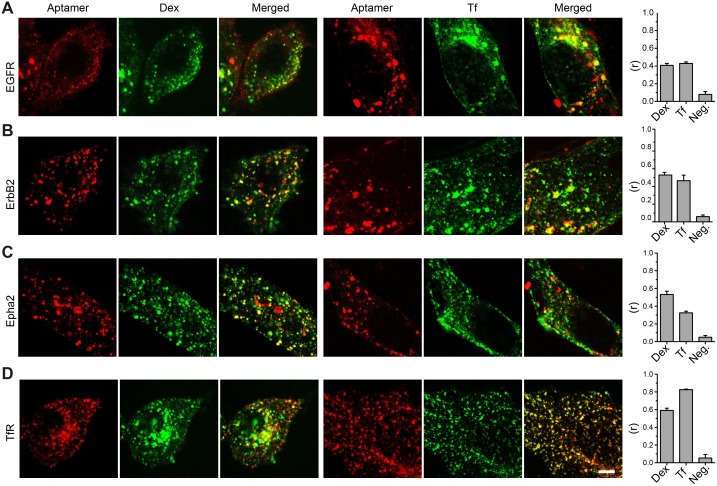
Co-localization of aptamers and classical endocytotic markers. (**A-C**) Confocal images of cells co-stained with aptamers and Alexa488-dextran (Dex) or Alexa488-Transferrin (Tf). (**D**) Confocal images of a validated aptamer against the transferrin receptor (TfR) [7] on HEK293 cells was used as positive co-localization control with Tf. Pearson’s correlations coefficient (r) were calculated from 3 independent experiments (10 to 20 cells analyzed per experiments). The negative control (Neg.) was obtained by flipping one of the images horizontally before computing the correlation coefficient. Bars represent the average of three independent experiments and error is the SEM. Scale bar represents 5 μm.

In order to demonstrate that the structures labeled by aptamers are intracellular organelles, we decided to study if the signals of aptamers co-localized with classical endocytic markers like Dextran (Dex) and Transferrin (Tf) [28]. Living cells were co-incubated with fluorescent aptamers and Alexa488-Dex or Alexa488-Tf. Qualitative inspection of laser confocal images suggests partial co-localizations between aptamers and Tf or Dex, which shows that aptamers reach different intracellular compartments (e.g. endosomes and lysosomes). Pearson´s correlation coefficient analysis provided the quantitative view of the co-localization between aptamers and the endocytic markers (Fig 2). In order to bring these correlation values in perspective, we also included a positive control. This was achieved by incubating our previously validated aptamer against transferrin receptor (TfR) with Alexa488-Tf [7]. If a correlation value of 0.8 was achieved using an aptamer binding to the TfR and its natural ligand Tf, values of 0.4 suggest a rather good co-localization considering that Dx and Tf are not the natural ligands of EGFR, ErbB2 or Epha2. More importantly, these results corroborate that the cellular structures stained with the fluorescent aptamers are intracellular endosome-like organelles.

### Antibody staining results in cellular fluorescence levels that are in the same range with the aptamer stainings

**Fig 3:**
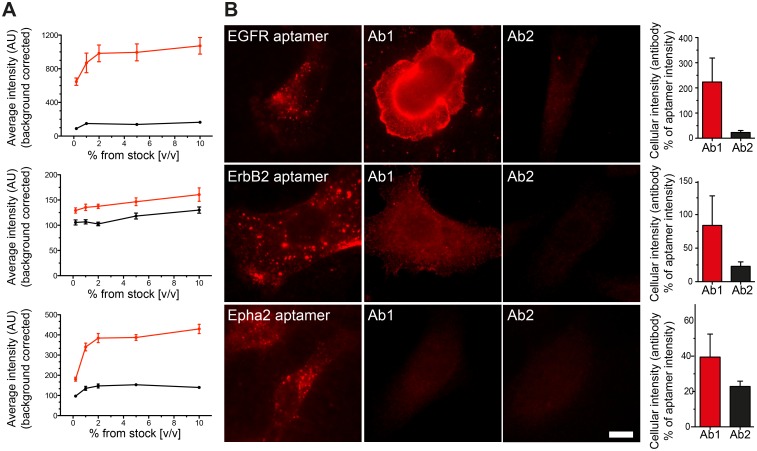
Direct comparison between aptamer and antibody stainings. **(A)** Antibody concentration was first titrated to perform optimal live-stained as explained in Fig 1A (Ab1: red trace and Ab2: black trace). Every point in the saturation curves represents the average intensity from 20 to 50 cells imaged with an epifluorescence microscope from at least 3 independent experiments (associated error is displayed as the SEM). All further antibody stainings through out the manuscript were used at 1% v/v (from the original stock concentration provided by the supplier). (**B**) Equally scaled images to allow a direct comparison between the staining pattern, performance and fluorescence intensity of cells stained with aptamers or antibodies. Scale bar: 5 μm. The bar graphs at the right of each panel show the average intensity of cells stained with antibodies (Ab1 and Ab2) expressed in percentage of the aptamers intensities. Error bars are the SEM from 3–4 independent experiments with more than 30 cells per experiment. Detailed information of all Ab1 and Ab2 antibodies can be found in Table 1 in the Materials and Methods section.

For every receptor type, we compared the fluorescence intensity obtained from cells live-stained with aptamers or live-stained with 2 different primary antibodies (see Table 1 in Materials and methods). We chose antibodies that were described to recognize epitopes at the extracellular domain of the receptor to allow us to perform the same live stainings like for the aptamers. After titrating the right amounts of antibody needed for the live immunostaining, we selected an antibody concentration that gave a saturating staining with minimum background (Fig 3A). The best working dilution was typically 1:100 (1% v/v from the original stock concentration obtained from the supplier) for all the antibodies tested. To achieve a proper antibody staining titration, all samples were imaged using an epifluorescence microscope to integrate the fluorescence signal from most of the cellular volume. Interestingly, different antibodies against the same target molecule performed very different in our titration assays. When compared the average cellular intensity of cells stained with aptamers or antibodies, most of the aptamer stainings resulted in brighter cells. The Ab1 against EGFR was the only exception generating cells ~100% brighter (i.e. ~2 fold) than if stained with the EGFR aptamer (Fig 3B)

**Table 1:** List of antibodies used.

Receptor name	Catalogue number	Company	Species, clonality	Name given throughout the study
EGFR	E2156	Sigma	Mouse, monoclonal	Ab1
EGFR	mAb4267	Cell Signaling	Rabbit, monoclonal	Ab2
ErbB2	Ab16899	Abcam	Mouse, monoclonal	Ab1
ErbB2	Ab135376	Abcam	Rabbit, monoclonal	Ab2
Epha2	Ab13770	Abcam	Rabbit, polyclonal	Ab1
Epha2	mAb6997	Cell Signaling	Rabbit, monoclonal	Ab2

### The fluorescence intensity of single antibodies is several-fold higher than that of single antibodies

**Fig 4:**
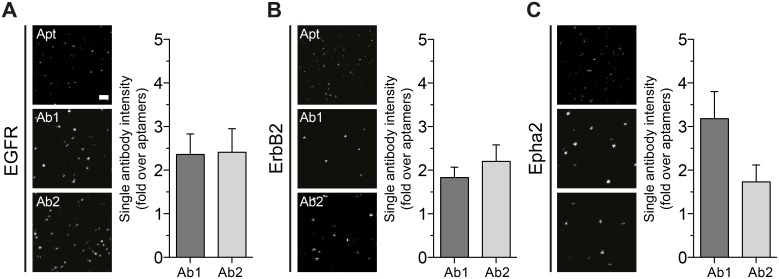
Average intensity of single antibodies and aptamers. **(A-C)** Examples of comparable STED images with single aptamers or single antibody packages (primary-secondary) seeded on glass coverslips. Scale bar (top image on panel A) represents 500 nm. The plots associated display the average intensity from more than thousand spots of single antibody packages expressed as fold of single aptamer intensity. Error bars are the SEM of 3 independent experiments.

A single aptamer binds only to one target molecule and it is coupled to exactly one fluorophore (see Materials and methods for coupling details). Therefore, aptamer stainings lack the fluorescent signal amplification obtained when using primary and secondary antibody detection, This stoichiometry feature of aptamers has been exploited to perform quantitative super-resolution imaging [29]. However, to have a more equivalent comparison we decided to measure the average intensity of a single aptamer and a single package of primary-secondary antibody. For this we measured the average intensity of spots on glass coverslips sparsely coated with aptamers or antibodies. The probe samples were highly diluted and sonicated to ensure the presences of monomers (see Material and methods for more details). The samples were imaged using stimulated emission depletion (STED) microscopy. Under these conditions single spots represent either a single aptamer or a single primary-secondary package (Fig 4). After analyzing several hundreds of spots, aptamers showed a consistent average intensity with a narrow distribution supporting their mono-fluorophore behavior. However, regardless of their clonality or species (poly- or monoclonal and rabbit or mouse, see Table 1) most of the primary-secondary antibody packages were in average ~2 or more times brighter than the average intensity of a single aptamer (Fig 4).

### Antibodies typically reveal fewer cellular epitopes than aptamers

**Fig 5:**
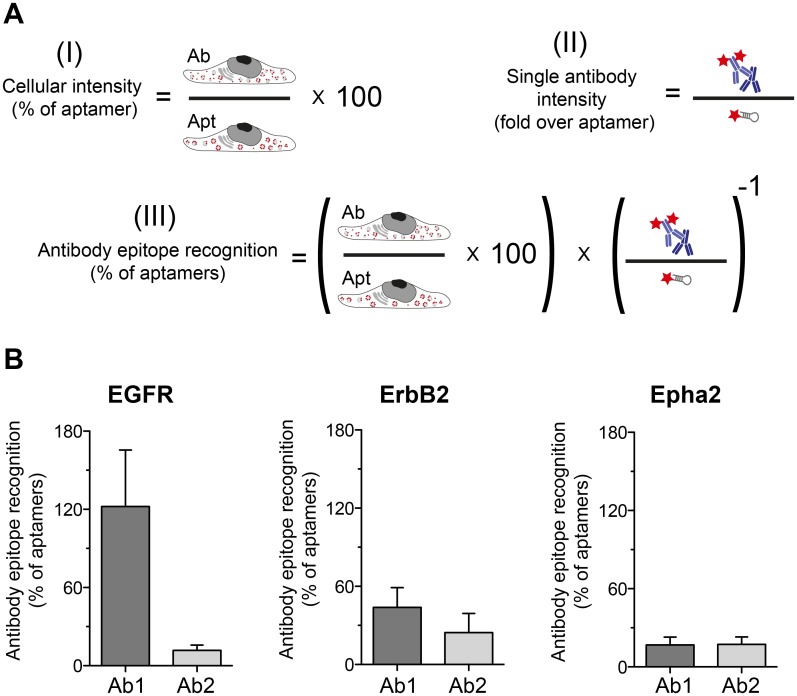
Estimations of the ability of antibodies or aptamers to recognized their epitopes. **(A)** Scheme explaining how the epitope recognition estimation was calculated. (I) Cellular intensity (% of aptamer) is the data also displayed in Fig 3B. (II) Intensity of single antibody package expressed as fold over the aptamer intensity is the data on Fig 4A–4C. (III) If we know the signal contribution of a single antibody package to the total cellular staining, we can estimate how many epitopes were found by the antibodies in relation to the epitopes found by the aptamers. (**B**) The results of the equation (III). Error bars represent the SEM from at least 3 independent experiments.

The fact that single antibody packages are in average ~2 folds brighter than a single aptamer brings a different perspective on the cellular intensities results shown in Fig 3. Why cells stained with aptamers are brighter than cells stained with antibodies if antibodies are in average ~2 folds brighter? We believe that similar to what was shown in previous investigation using other small probes [5,6], the smaller probes can provide a higher density of labeling. In other words small probes are able to find more epitopes than bulky antibody packages. We use our data from Figs 3 and 4 to estimate how much epitopes are antibodies or aptamers able to find (scheme in Fig 5A). With the exception of Ab1 for EGFR, which showed a similar proportion of detected epitopes as the EGFR aptamer, all other antibodies detected ~50% or less epitopes than their respective aptamer (Fig 5B).

### The higher epitope labeling density provided by the aptamers results in better structure recognition

Incomplete decoration of the protein of interest in an organelle by the affinity probe will typically result in spotty patterns in super-resolution microscopy [6,7,30]. Interestingly, a poor labeling of abundant and round structures like endosomes generates a high number of spots that makes it difficult to discern to which of the round structures a particular spot belongs to. The tendency of aptamers to find more epitopes, as shown in Fig 5, might result in a more continuous decoration of the stained structures. To check this hypothesis, we tested the capacity of antibodies or aptamers to reveal the structures of endosome-like organelles using super-resolution microscopy.

**Fig 6:**
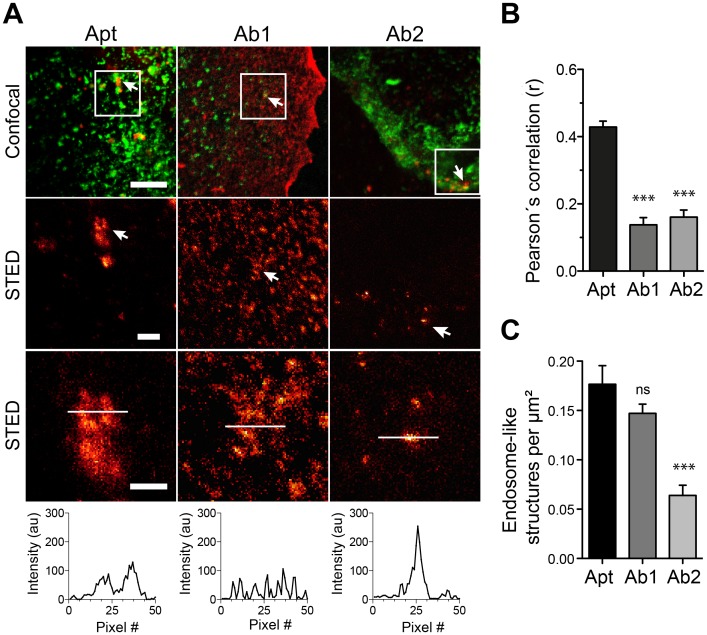
Recognition of subcellular structures using STED microscopy in aptamer or antibody stained samples against EGFR. **(A)** Sets of confocal and STED images of aptamers (Apt, red) or the antibodies Ab1 and Ab2 (red) co-stained with Alexa488-Transferrin (green). The first row of images shows confocal co-localization examples. The following rows of images show STED examples at different zooms from the white square delineated on the confocal images. A line scan of 50 pixels was drawn (white line) on the endosome-like structures. The line-scan intensity profile is displayed. (**B**) Pearson’s correlation coefficients (r) on confocal images obtained after staining with aptamers or antibodies with the Alexa488-Transferrin marker. (**C**) Quantification of endosome-like structures found in STED images for aptamer stainings (Apt) and for the antibodies (Ab1 & Ab2). All graphs (for B and C) show the mean values and S.E.M of 3 independent experiments. Statistical significances were calculated using one-way *Anova* and Dunnett’s multiple comparisons post hoc tests (ns, not significant; * p < 0.05; ** p < 0.01; *** p < 0.001). Scale bars from top to bottom row: 5, 1 and 0.5 μm.

**Fig 7:**
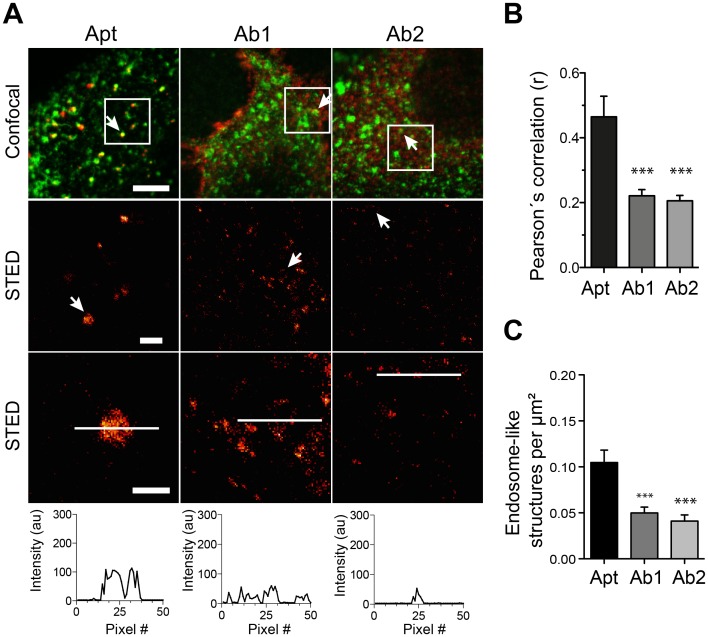
Recognition of subcellular structures using STED microscopy in aptamer or antibody stained samples against ErbB2. Figure legend is equivalent to legend on Fig 6, but for ErbB2.

**Fig 8:**
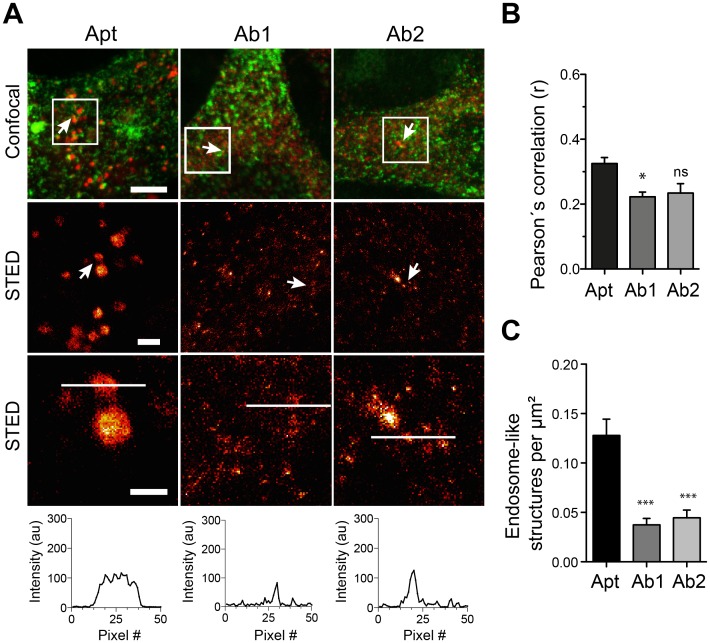
Recognition of subcellular structures using STED microscopy in aptamer or antibody stained samples against Epha2. Figure legend is equivalent to legend on Fig 6, but for Epha2.

We then turned to image these structures using STED microscopy when stained by aptamers or by the respective antibodies together with the general endocytic marker Tf as guidance (Figs 6, 7 & 8). Line scans on zoomed organelles detected by aptamers or antibodies in STED mode helped reveal, at first qualitatively, the differences in decoration of organelle if using aptamers or antibodies (Figs 6A, 7A & 8A). Additionally, we measure the Pearson´s correlation coefficient “r” between aptamers or antibodies with the Tf marker (from confocal images). Interestingly, the correlation between Tf and the antibodies staining was significantly poorer than between aptamers and Tf (Figs 6B, 7B & 8B). This again supports the inferior staining performance of most of the antibodies here tested. However, to quantify the differences observed between aptamers and antibody stainings we chose two random areas for each imaged cell and endosome-like structures were manually counted. Our quantifications suggest that endosomes-like structures stained with aptamers were typically easier to recognize (and count) than those stained with antibodies (Figs 6C, 7C & 8C). It is important to notice that the best antibody against EGFR (Ab1) display an equivalent capacity to reveal endosome-like structures (Fig 6C). This is in accordance with the epitope recognition capability shown in Fig 5, in which only this antibody provided comparable epitope recognition to the corresponding aptamer.

## Discussion

Here we have compared some staining features of commercial aptamers and 6 different antibodies (two per aptamer target). We incubated the cells with aptamers or antibodies for a rather long period of time (60 min) to maximize the labeling of intracellular organelles (endocytosed receptors), but not to study the peculiarities or endocytic mechanism of each of the receptors targeted by the affinity probes here tested. Our main observation was that aptamers tend to find more epitopes in crowded cellular environments (Fig 5). This also resulted in a better description of the endosome-like cellular structures that contained the target molecules (Figs 6, 7 & 8). As suggested in previous studies, the use of small affinity probes like aptamers [7] and nanobodies [5,6] has several advantages in light microscopy, especially in the super-resolution field. Small probes are less impaired by steric hindrance, allowing them to penetrate biological samples easily [31] and find epitopes which are not accessible by larger antibodies [32]. Therefore, the suboptimal performance of most of the antibodies here tested can be attributed to their large size, which hampers their diffusion and consequently their ability to find epitopes. On the other hand, the small size of aptamers confers them the ability to move more, with fewer steric restrictions and thus finding more target molecules. Interestingly, one of the antibodies here tested (Ab1 for EGFR) was able to find a similar amount of epitopes as the aptamer (Fig 5B). This finding reveals the importance of how buried or hidden the epitope is, and how variable can be results from different antibodies against the same target (e.g. Ab1 vs Ab2 of EGFR). For instance, it seemed that the Ab1 antibody has a similar access to EGFR than its aptamer. Under the Ab1 for EGFR circumstance the antibodies can provide a clear decoration of the endosome-like structures and even a brighter staining due to the amplification of signal generated by the use of secondary antibodies (Fig 4). In summary, although the fluorescence intensity of a single aptamer is typically lower than for an average primary/secondary antibody package, their small size allows them to find more target molecules, resulting in brighter images. However, the brightness of the signal will also depend on the abundance of target molecules and as mentioned on the epitope accessibility. In this study we targeted extracellular receptors, which might be in general more accessible for affinity probes than targets within the crowded cytosol.

The size of the affinity probe does not only play a role for the ability to find epitopes, but is also crucial for positioning the fluorophore with respect to the target molecule [6]. This linkage inaccuracy is of much relevance since the newer super-resolution techniques are reaching precisions comparable or smaller than the size of an antibody package [1,4]. In an ideal approach, the fluorophores would be placed directly on the target molecules, without the need of an affinity probe. This could be achieved by bioorthogonal conjugation chemistry. However, the most common click chemistry method for cellular proteins labels all proteins [33,34] and only recently a laborious strategy has appeared to target the click reaction to a particular protein of interest [35]. Therefore, the generation and characterization of small binders like aptamers that place the fluorescent moiety between 1 and 4 nm from the intended target are of major relevance in the rather new field of light microscopy of biology at the nanoscale. Additionally, the accurate positioning of the dye together with the fact that aptamers are equipped with only one fluorophore and bind only one target molecule have recently been exploited to perform quantitative super-resolution imaging [29].

Although it is possible to find aptamers that bind selectively to fixed targets in tissue samples [36] it has been difficult to select for aptamers that bind aldehyde fixed proteins. The selection of aptamers that recognize formaldehyde fixed proteins has not been studied extensively. Therefore, most of the available aptamers bind to extracellular epitopes and thus aptamers can be applied in living cells where the epitope is natively folded and without perturbing chemical modifications. It is important to mention that antibodies can also be affected by the fixation chemistry, which is indeed the case of most antibodies if using glutaraldehyde as fixative. Nevertheless, antibody sensitivity to aldehyde modifications is drastically less than for aptamers. Therefore, the use of aptamers as staining tools still needs further development, especially in the selection of aptamers able to bind cytosolic and chemically fixed target proteins. Despite these shortcomings, our data support the conclusion that aptamers as small affinity binders can deliver accurate and in principle quantitative images for cell biology. The recent rise of commercially available aptamers should be considered as a new gamut of affinity probes to use in microscopy, in particular for current super-resolution techniques.

## Materials and methods

### Materials

Chemicals, buffers, and other reagents were purchased from Sigma–Aldrich (Seelze, Germany) unless otherwise specified. Cell culture media RPMI 1640 and DMEM-High glucose (4.5 g/L) Dulbecco’s modified Eagle’s were from Life Technologies, Inc. (Grand Island, NY) and Invitrogen^™^ by Life Technologies (Carlsbad, USA), respectively. Fetal calf serum (FCS) was from PAA laboratories GmbH (Pasching, Austria), Penicillin/streptomycin 10.000 U/ml each, L-Glutamine 200 mM and Trypsin-EDTA solution were purchased from Lonza (Basel, Switzerland). For aptamer staining, sheared salmon sperm DNA (10 mg/ml) was purchased from Invitrogen^™^ by Life Technologies (Carlsbad, USA).

### Cell culture

HeLa (Epha2a positive), MCF7 (EGFR and ErbB2 negative) and HEK293 (Epha2a negative) cell lines were cultured in complete DMEM medium containing 10% FCS, 4 mM L-Glutamine and 100 U/ml each penicillin and streptomycin. SKBR3 (ErbB2 positive) and A-431 (EGFR positive) cells were cultured in complete RPMI medium supplemented with 10% FCS, 4 mM L-Glutamine and 100U/ml each penicillin. For imaging, cells were seeded into 12 well plates containing PLL coated coverslips and incubated at 37°C in a humid atmosphere with 5% CO_2_ until they reached 70–80% confluence. A-431 cells were obtained from the American Type Culture Collection (ATCC) and all other cells lines were purchased from the Leibniz Institute DSMZ-German Collection of Microorganisms and Cell Cultures.

### Fluorophore coupling to aptamers

Unconjugated DNA aptamers containing C6-Thiol group at the 5´-end and the 5-BzdU modifications were developed by Aptamer Sciences, Inc. (Pohang, South Korea) and supplied by AMS Biotechnology (Europe). The aptamers used in this study were against EGFR (5'-SH-EGFR aptamer-3’, seq # 2369-27-02, 50mer), ErbB2 (5'-SH-ErbB2 aptamer-3', seq # 1194–35, 40mer) and Epha2 (5'-SH-EphA2 aptamer-3', seq # 2176-01-01, 76mer). The sequences of the aptamers and the location of their 5-BzdU modifications are not revealed by the supplier. However, target specificity experiments were provided by the supplier and reproduced in this study (Fig 1).

Thiol-maleimide cross-linking reaction was performed to conjugate the ATTO 647N fluorophore to the aptamers (ATTO-TEC GmbH Siegen, Germany). A thiol modified aptamer (10 nmol) was reduced using 10 mM TCEP dissolved TEAA (1 M Triethylammonium acetate buffer pH 7.0), heated for 3 minutes at 70°C followed by 60 minutes incubation at room temperature. After desalting reduced aptamers quickly into Dulbecco´s PBS (DPBS) using Biospin 6 columns, approximately 10 nmol of the aptamer was mixed with 4 molar excess of maleimide-functionalized fluorophore ATTO 647N (dissolved in anhydrous DMSO to a concentration of 10 μg/μl). This coupling reaction was incubated overnight at 4°C in dark conditions and then recovered by ethanol precipitation. Alternatively, reduction of the disulphide was performed with 100 mM DTT at 37°C for 1h, followed by extraction with EtOAc and precipitation with ethanol. The pellet was dissolved in PBS, and conjugation with ATTO 647N maleimide was successfully achieved upon incubation at 37°C for 3h at a dye concentration of 1 mM. In both protocols, excess of chromophore was removed by extraction and precipitation, followed by desalting using a Biospin 6 column. The labelling efficiency was analysed by anion exchange HPLC under denaturing conditions (6 M urea, 70°C). Aliquots of the labelled aptamers were run on a Dionex DNAPAc PA200 column and absorbance was monitored at 260 nm and 650 nm. For each aptamer, 30 μM stock solutions were prepared and stored protected from light at -20°C until use.

### Live staining of cells with aptamers

To ensure the proper folding of the fluorescently labeled aptamers, a solution containing 10 μM of aptamer in 5x phosphate buffered saline with 5 mM MgCl_2_ was heated up to 75°C for 3 minutes and then cooled down to 20°C at a rate of 1°C/minute using a thermal cycler. To perform the cellular staining, cells seeded in a glass coverslips were briefly washed with complete DMEM medium and incubated with blocking solution (complete DMEM supplemented with 100 μg/ml sheared salmon sperm DNA and 1 mM dextran sulphate) for 10 minutes at 37°C and 5% CO_2_. Subsequently, coverslips were carefully incubated upside down with 60 μl of staining solution (complete DMEM supplemented with 100 μg/ml sheared salmon sperm DNA and 250 nM folded aptamer) for 1 hour at 37°C and 5% CO_2_. After incubation, coverslips were washed in large volume of ice-cold Dulbecco´s PBS (DPBS) and cells were fixed first with ice-cold 4% PFA on ice for 20 minutes and then moved to room temperature for extra 25 minutes. After quenching remaining aldehydes (0.1 M glycine in DPBS) for 15 minutes and washing with twice with DPBS (5 minutes each) the coverslips were mounted in Mowiol (6 g glycerol, 6 ml deionized water, 12 ml 0.2 M Tris buffer pH 8.5, 2.4 g Mowiol^®^ 4–88, Merck Millipore), dried and stored at 4°C in the dark until imaging. To determine the saturating concentration for the 60 minutes stainings (~250 nM), aptamers were tested as described above but using 10, 100, 250, 500 & 1000 nM final concentrations (Data not shown).

### Live staining of cells with antibodies

For each receptor analyzed, two different primary antibodies were tested (Table 1). All antibodies used bind extracellular epitopes of their target receptors. Secondary antibodies conjugated to ATTO 647N, goat anti-mouse IgG (# 610-156-121) and goat anti-rabbit IgG (# 611-156-122) were from Rockland Immunochemicals Inc. (Gilbertsville, PA, USA).

Cells on coverslips were briefly washed with complete DMEM medium and incubated with blocking solution (complete DMEM supplemented with 2% bovine serum albumin) for 10 minutes at 37°C and 5% CO_2_. Coverslips were carefully removed and incubated for 1 hour with primary antibody (1:100) in complete DMEM at 37°C and 5% CO_2_. After incubation, coverslips were washed with large volume of ice-cold DPBS and cells were then fixed with ice-cold 4% PFA on ice for 20 minutes followed by 30 minutes at room temperature. Remaining aldehydes were quenched for 15 min with 0.1 M glycine in DPBS. Mild permeabilization was performed for 10 min using DPBS supplemented with 2% BSA and 0.1% Triton X-100. Secondary antibodies (1:500) were incubated for 60 minutes at room temperature in a humid chamber protected from light. After incubation, cells were thoroughly washed with DPBS and mounted in Mowiol. Slides were stored at 4°C in the dark until imaging.

### Co-localizations and endosome-like structures identification

For co-localization experiments cells were co-incubated with 250 nM folded aptamers and 50 μg/ml of Alexa488-transferrin (Invitrogen^™^ by Life Technologies; Carlsbad, MA, USA) or 200 μg/ml Alexa488-dextran (Thermo Fisher Scientific Inc. Waltham, MA, USA). Subsequent washings, fixations and quenching steps were performed as described above.

For identification of endosome-like structures a co-staining was performed using aptamers or antibodies and Alexa488-transferrin. Cells were co-incubated with primary antibodies or aptamers as described above with 50 μg/ml Alexa488-transferrin for 1 hour at 37°C and 5% CO_2_ in complete DMEM. Washing, fixation, permeabilisation and incubation of secondary antibodies were performed as described above.

### Imaging

Conventional epifluorescence images were obtained with an Olympus IX71 microscope equipped with 0.75 NA/60x oil objective and captured with an Olympus F-View II CCD camera (Olympus, Hamburg, Germany) (Used for Fig 1 and cellular intensity analysis on Fig 3). Confocal (Figs 2, 6, 7 & 8) and STED (Figs 4, 6, 7 & 8) images were obtained with a True Confocal System STED SP5 fluorescence microscope (Leica Microsystems GmbH, Mannheim, Germany) equipped with a 1.4 NA/100x STED objective. For confocal imaging, pixel size was set to 50.5 nm and the scanning speed to 1 kHz. Signal was detected with using photomultipliers (PMTs). Excitation was performed with a 633 nm helium/neon laser line and the signal was detected between 640 and 730 nm. For STED imaging, pixel size was set to 20.2 nm and scanning speed to 1 kHz. Signal was detected with an avalanche photodiode detector (APD). Excitation was performed with a 640 nm diode laser and depletion with a MaiTai pulsed tunable laser at 750 nm (Mai Tai Broadband, Spectra-Physics, Santa Clara, CA, USA).

### Data analysis and statistics

Data analyses on Figs 3, 4, 5, 6, 7 and 8 were performed with custom-written procedures in Matlab (MathWorks Inc., Massachusetts, USA). ROI selection for cells: An experienced user selected manually the outline of each cell, and also a background region, in the vicinity of the respective cell. The average signal intensity within the cellular region of interest was calculated, and was corrected for the background intensity by subtracting the average signal intensity from the background region of interest. STED spots in Fig 4: An experienced user selected manually a region of interest containing numerous individual spots, and a neighboring region containing only background signals. The region of interest was subjected to a median filter, which removed single pixel noise (“salt-and-pepper” noise). The signals that were superior to the average background signal were then identified by an automatic thresholding procedure, relying on a user-defined threshold (4 arbitrary units above the mean intensity value of the background). The centers of the spots with intensities above background were then identified, and line scans were performed in both the vertical and horizontal directions. Lorentzian curves were fit onto the line scans. The fits provided the peak intensities of the spots. Only fits that resulted in a root mean square deviation of less than 5% of the signal, for both the vertical and horizontal directions, were accepted in the measurements. Counting of endosome-like structures in Figs 6, 7 & 8 was performed by manual inspections of 2 regions of 26.7 μm^2^ per cell. Pearson´s correlation coefficient (PCC) “r” was obtained using the ImageJ plug-in “Manders Coefficients”. For the actual analysis, individual cells were selected (ROI) to avoid calculating PCC from areas without cells/signal (which tent to inflate the PCC value), All images for PCC analysis were obtained using the same imaging conditions (e.g. laser power, PMT gain, pinhole, etc) and no threshold was applied for the PCC calculation. Graphs and statistical analysis were carried out using custom-written procedures, in Matlab (MathWorks Inc., Massachusetts, USA), Sigma Plot (Systat Software, San Jose, CA, USA) or GraphPad Prism 5.0 (San Diego, CA, USA). All values are given as mean ± standard error of the mean (SEM) from at least 3 independent experiments. Statistical comparisons were performed using GraphPad Prism 5.0 and are described in the Figure legend.
